# TWEAK/Fn14 and Non-Canonical NF-kappaB Signaling in Kidney Disease

**DOI:** 10.3389/fimmu.2013.00447

**Published:** 2013-12-10

**Authors:** Jonay Poveda, Luis C. Tabara, Beatriz Fernandez-Fernandez, Catalina Martin-Cleary, Ana B. Sanz, Rafael Selgas, Alberto Ortiz, Maria D. Sanchez-Niño

**Affiliations:** ^1^Department of Nephrology, IIS-Fundacion Jimenez Diaz, Universidad Autonoma de Madrid and IRSIN, Madrid, Spain; ^2^Department of Nephrology, IdiPAZ, Madrid, Spain

**Keywords:** acute kidney injury, fibrosis, inflammation, kidney, lupus nephritis, podocyte, proteinuria

## Abstract

The incidence of acute kidney injury (AKI) and chronic kidney disease (CKD) is increasing. However, there is no effective therapy for AKI and current approaches only slow down, but do not prevent progression of CKD. TWEAK is a TNF superfamily cytokine. A solid base of preclinical data suggests a role of therapies targeting the TWEAK or its receptor Fn14 in AKI and CKD. In particular TWEAK/Fn14 targeting may preserve renal function and decrease cell death, inflammation, proteinuria, and fibrosis in mouse animal models. Furthermore there is clinical evidence for a role of TWEAK in human kidney injury including increased tissue and/or urinary levels of TWEAK and parenchymal renal cell expression of the receptor Fn14. In this regard, clinical trials of TWEAK targeting are ongoing in lupus nephritis. Nuclear factor-kappa B (NF-κB) activation plays a key role in TWEAK-elicited inflammatory responses. Activation of the non-canonical NF-κB pathway is a critical difference between TWEAK and TNF. TWEAK activation of the non-canonical NF-κB pathways promotes inflammatory responses in tubular cells. However, there is an incomplete understanding of the role of non-canonical NF-κB activation in kidney disease and on its contribution to TWEAK actions *in vivo*.

## Unsolved Issues in Kidney Disease

Acute kidney injury (AKI) and chronic kidney disease (CKD) are the most severe forms of kidney disease (1, 2). AKI is characterized by a sudden loss of renal function. AKI patients have increased short- and long-term mortality and risk of CKD progression. However, there is no therapy that accelerates recovery from AKI. CKD is a major healthcare problem, with more than 20 million aged 20 years or older affected in the United States. Diabetic kidney disease is the leading cause of end stage renal disease in the Western Countries. However, current treatments based on blockade of the renin-angiotensin system are not sufficient to prevent progression of diabetic kidney disease (3).

**Figure 1:**
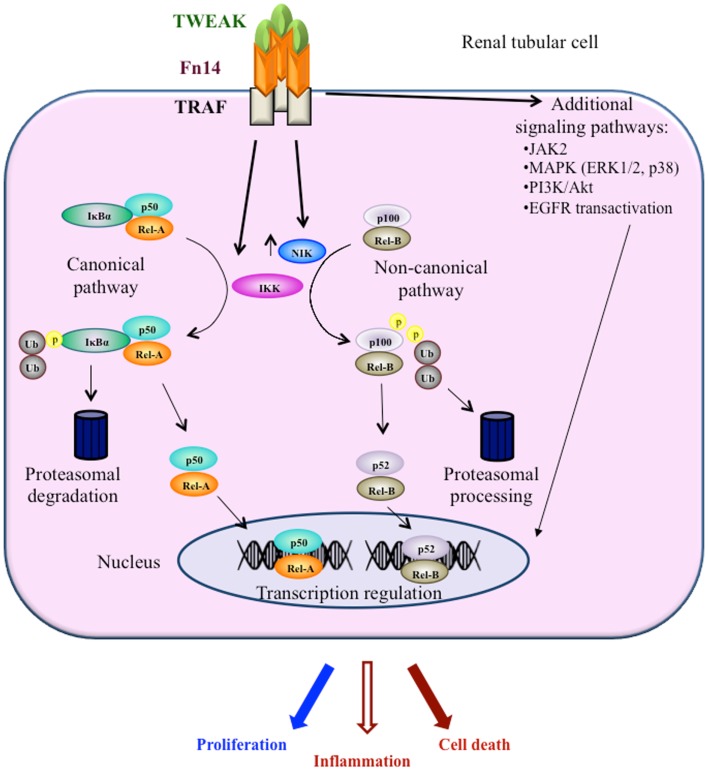
**Key intracellular pathways activated by TWEAK engagement of Fn14 in kidney tubular cells**. TWEAK signaling in kidney cells has been characterized most in detail in tubular cells. TWEAK engages both the canonical and the non-canonical NFκB pathways and kinase signaling mechanisms.

Recent evidence suggests a role for TNF superfamily member Tumor necrosis factor-like weak inducer of apoptosis (TWEAK, Apo3L, or TNFSF12) in both AKI and CKD, where it has been shown to regulate cell death, inflammation, and fibrosis through activation of the TWEAK receptor Fn14 and a variety of intracellular signaling pathways, including the transcription factor nuclear factor-kappa B (NF-κB) (4, 5) (Figure 1). Clinical trials are testing anti-TWEAK neutralizing antibodies^1^^,^^2^. One key difference between TWEAK and the best characterized member of the family, TNF, is that TWEAK activates the non-canonical NF-κB pathway. We now review current information on TWEAK, non-canonical NF-κB activation, and kidney disease.

## Tweak

TWEAK may be membrane-bound or soluble, although most functional studies have been performed with soluble TWEAK. Soluble TWEAK is thought to be generated from full-length TWEAK by furin-mediated cleavage of the extracellular domain (6).

The TWEAK receptor, Fn14 (TNFRSF12a), is the smallest member of the TNF receptor superfamily. Fn14 is a type I transmembrane protein which has 102 aa in its mature isoform. The extracellular domain has 53 aa and harbors a cysteine rich domain required for TWEAK binding (7). Interestingly, the Fn14 intracellular domain (29 aa) lacks the characteristic death domain of TNFRSF receptors but contains TNFR-associated factor (TRAF) binding sites. Fn14 trimerization recruits TRAF2 and TRAF3 upon TWEAK binding (8).

TWEAK may regulate cell proliferation, cell death, cell differentiation, and inflammation (4, 6).

TWEAK may trigger cell death or proliferation processes, depending on cell type and microenvironment; TWEAK promotes proliferation of numerous cell types including quiescent renal tubular cells through activation of NF-κB, MAPK, and phosphatidyl-inositol 3-kinase (PI3K)/AKT pathways (9). In addition TWEAK was described as a weak inductor of apoptosis which required special microenvironment (such as the presence of interferon-γ – IFN-γ) to induce cell death (10–12). Under certain circumstances TWEAK can induce apoptosis without co-treatment with other cytokines. It has been proposed that levels of Fn14 expression may sensitize cells to TWEAK but it is also clear that this cannot be the only mechanism (9). Indeed, the signaling cascade which triggers cell death following Fn14 activation remains poorly understood as Fn14 does not contain a death domain (7). Induction of TNF expression by TWEAK has been reported in certain cell types. In immortalized tumor cells, TWEAK activation of Fn14 recruits a TRAF2/cellular inhibitor of apoptosis 1 (cIAP1) complex that results in the lysosomal degradation of cIAP1-TRAF2 in a cIAP1-dependent manner (13). TWEAK depletion of cIAP1 and TRAF2 activates non-canonical NF-κB signaling. However the function of non-canonical NF-κB signaling was not explored.

TWEAK is expressed in many tissues. High levels are found in the pancreas, intestine, heart, brain, lung, ovary, vasculature, and skeletal muscle, and lower levels in the liver and kidney (4). Fn14 is expressed by many cell types, including epithelial, mesenchymal, and endothelial cells. In healthy tissues Fn14 expression is low. However, cellular Fn14 levels are increased in response to stress or injury.

## TWEAK/Fn14 in Kidney Disease

Fn14 is expressed in kidney tubular cells, mesangial cells, and podocytes (10). Fn14 expression by kidney endothelium has not been well characterized. Renal infiltrating cells such as macrophages also express Fn14 (14). Fn14-expressing cells are potentially responsive to TWEAK. In addition TWEAK and Fn14 expression is increased in kidney injury and targeting of the system is beneficial in different models of kidney injury.

### TWEAK/Fn14 actions on renal cells

Potential kidney sources of TWEAK include infiltrating monocytes and T lymphocytes and local cells such as mesangial and tubular cells (10, 15–17).

During glomerular injury both mesangial cells and podocytes may be targets of the inflammatory response. Mesangial cell injury is observed in proliferative glomerulonephritis, while podocyte injury is characteristic of proteinuric kidney diseases. TWEAK promotes the expression of chemokines, adhesion molecules, and matrix metalloproteinases in human and murine mesangial cells (17, 18). TWEAK also increases mesangial cells proliferation, but TWEAK combined with IFN-γ promotes mesangial cells apoptosis (17, 18). In human and murine podocytes TWEAK induces the expression of proinflammatory mediators in an NF-κB-dependent manner (18, 19). TWEAK also promotes nephrin expression and human podocyte proliferation (18). Expression of nephrin and proliferation are not usually associated *in vivo*. In fact, podocytes are terminally differentiated cells that do not divide. Podocyte proliferation is only observed under very specific pathological circumstances and is usually associated with dedifferentiation and loss of podocyte markers including nephrin.

In murine and human renal tubular cells TWEAK also promotes the expression of cytokines and chemokines (20). TWEAK also increases tubular cell proliferation through recruitment of the mitogen-activated protein kinases ERK and p38, the PI3K/Akt pathway and the canonical NF-κB pathway (9). Similar to observations in mesangial cells, in a proinflammatory milieu TWEAK induces apoptosis of tubular cells (10). By contrast to mesangial cells, the lethal action of TWEAK in tubular cells requires the simultaneous presence of TNFα and INFγ. Surprisingly, caspase inhibition prevented the features of apoptosis induced by the cytokine cocktail but increased overall cell death through a reactive oxygen species-dependent necrotic pathway (10). More recently, TWEAK/TNFα/INFγ-induced cell death in tubular cells was shown to have features of necroptosis (21). Necroptosis is an active form of cell death that requires the kinase activity of receptor-interacting protein 1 (RIP1) and RIP3.

TWEAK also promotes murine renal fibroblasts proliferation through activation of the Ras/ERK pathway (22). The proliferative effect of TWEAK on fibroblasts overrides its negative effect on extracellular matrix production. Thus, the overall effect of TWEAK targeting in experimental renal fibrosis is decreased fibrosis (22). In addition, TWEAK also promotes the expression of inflammatory cytokines in renal fibroblasts (22).

So far, the proinflammatory effect of TWEAK on mesangial cells, podocytes, and fibroblasts have been shown to proceed through canonical NF-κB activation involving RelA migration to the nucleus and expression of canonical RelA targets such as MCP1, RANTES, and others (18, 19, 22). By contrast, both canonical and non-canonical NF-κB activation by TWEAK have been observed in tubular cells (20, 23). The known consequences of non-canonical NF-κB activation are discussed below.

### TWEAK/Fn14 expression in kidney injury

TWEAK and Fn14 expression is increased in experimental animal models of AKI, lupus nephritis, albumin overdose-induced proteinuria, kidney fibrosis induced by unilateral ureteral obstruction and anti-GBM nephritis (10, 19, 20, 22, 24, 25). High levels of tubular Fn14 expression have been also observed in human ischemic AKI and in acute or chronic human tubulointerstitial inflammation (24, 26). In human lupus nephritis glomerular Fn14 mRNA expression was increased and was higher in proliferative than in membranous lupus nephropathy (27, 28). Urinary TWEAK has been proposed as a biomarker of lupus nephritis activity (29–32).

### Therapeutic modulation of TWEAK or Fn14 in experimental kidney injury

Therapeutic modulation of the TWEAK/Fn14 pathway has been successful in experimental models of AKI, kidney fibrosis, lipid-induced kidney injury, proteinuria-induced kidney injury, and immune-mediated glomerular injury, including lupus nephritis. The TWEAK/Fn14 pathway was modulated in mice either by gene targeting of TWEAK/Fn14, by neutralizing anti-TWEAK antibodies or by blocking anti-Fn14 antibodies.

Mice with experimental ischemic or folic acid-induced AKI displayed a variety of benefits from TWEAK targeting that included better histological parameters and renal function, and reduction of chemokine expression, tubular cell apoptosis, and renal fibrosis, while the anti-inflammatory and anti-aging hormone klotho was increased (4, 9, 20, 23, 24, 26, 33). TWEAK downregulates Klotho in normal kidneys (33).

Fn14-deficient mice show decreased kidney damage, inflammation, and fibrosis in models of lupus nephritis (5, 34). Anti-TWEAK neutralizing antibodies reduced inflammatory gene expression and renal damage in lupus nephritis (34). Reduced residual fibrosis was observed in mice which had been protected from the acute phase of ischemia reperfusion by anti-Fn14 blocking antibodies (24). Protection from fibrosis by interfering with TWEAK/Fn14 is not limited to residual fibrosis following amelioration of the initial injury. TWEAK knockout mice were protected from fibrosis in the unilateral ureteral obstruction of model of persistent kidney insult while overexpression of TWEAK causes renal fibrosis in normal previously normal kidneys (22).

Fn14-deficient mice were protected from anti-GBM induced glomerulonephritis (25). In addition, neutralizing anti-TWEAK antibodies improved nephritis in wild type mice without altering the adaptive immune response, indicating that TWEAK/Fn14 directly regulates the inflammatory response (25). In this regard, anti-TWEAK antibodies decreased hyperlipidemia-induced kidney inflammation and injury (35).

Experimental kidney diseases in which TWEAK/Fn14 targeting has been successful share the presence of diverse degrees of local inflammation. Thus, the kidney milieu to some extent reproduces the cell culture conditions under which TWEAK promotes kidney cell death. However, the environment also influences TWEAK actions in the kidney *in vivo*. The TWEAK/Fn14 pathway may contribute to tissue regeneration (9, 36, 37). In experimental, inflammation-free unilateral nephrectomy TWEAK promotes remnant kidney growth and tubular cell proliferation (9). However, TWEAK knockout mice have decreased remnant kidney size and tubular cell proliferation (9). This information may be useful in the context of regenerative medicine. However, the regenerative potential of TWEAK was not apparent in animal models of inflammatory kidney injury, where the injurious effect was observed in all models studied so far.

## Non-Canonical NF-κB Signaling

The NF-κB transcription factor binds to the κB enhancer in DNA to control transcription of over 400 genes. NF-κB controls immune and inflammatory responses, developmental processes, cellular growth, and apoptosis. Dysregulation of NF-κB has been linked to cancer, inflammatory, and autoimmune diseases (9, 23, 38).

The mammalian NF-κB family has five members, RelA/p65, RelB, c-Rel, NF-κB1 p50, and NF-κB2 p52 (39, 40). All share a highly conserved DNA-binding/dimerization domain called the Rel homology domain (RHD), through which they form homo or heterodimers. RelA, c-Rel, and RelB contain a C-terminal transactivation domain (TAD) with multiple ankyrin repeats. In order to activate transcription, they form dimers with either p50 or p52.

Nuclear factor-kappa B activation does not require the novo synthesis of NF-κB proteins. In most cells, NF-κB proteins are present as an inactive complex in the cytoplasm. The activity of NF-κB is regulated by its interaction with inhibitory IκB proteins. The IκB proteins include p105, p100, IkBα, IκBβ, IκBγ, IκBϵ, IκBz, and Bcl-3 (41–43), NFκB1 and NFκB2 are synthesized as precursors, p105 and p100, respectively. These precursors contain an IκB-like C-terminal portion and function as NF-κB inhibitors. Ubiquitin/proteasome processing results in selective degradation of the C-terminal ankyrin repeats, disrupts the IκB-like function and generates the active NF-κB subunits p50 and p52 (44, 45).

Nuclear factor-kappa B activation in response to extracellular signals can proceed through classical/canonical, alternative/non-canonical, or hybrid pathways (4, 38, 46–49). Classical NF-κB activation is a rapid and transient response to a wide range of stimuli, while the alternative pathway involves slow activation of the p100/RelB heterodimer leading to the generation of p52/RelB and prolonged activation of NF-κB target genes in response to a more limited set of stimuli (45, 50). There is interplay between both pathways. Thus, classical NF-κB activation-induced transcription of NF-κB2 and RelB favors activation of the non-canonical pathway. Both pathways converge on the activation of a complex that contains a serine-specific IκB kinase (IKK). IKK contains at least, three distinct subunits: the catalytic kinase subunits IKKα (IKK1) and IKKβ (IKK2) and the regulatory subunit, IKKγ (NEMO).

Nuclear factor-kappa B inducing kinase (NIK, MAP3K14) is the apical kinase triggering non-canonical NF-κB activation. NIK belongs to the family of MAP3Ks that are known to be activated through T-loop phosphorylation. Upon activation, NIK activates IKKα and serves as a docking molecule that recruits IKKα to p100, facilitating ubiquitination by the β-TrCP ubiquitin ligase and subsequent proteasomal processing into the mature p52 subunit in a manner dependent on IKKα-dependent p100 phosphorylation (50–52). This allows the RelB/p52 heterodimer to translocate to the nucleus and to activate transcription of target genes (53). p100 processing is regulated by a short list of activators known to signal through NIK (53–57). This list includes TWEAK (58).

A variety of functions have been described for NIK including generation and/or maintenance of memory T cells (59), the formation of Th17 cells (60), promotion of glucagon responses (61), and the pathogenesis of chronic inflammation and insulin resistance in type 2 diabetes (62). Some of these functions may be independent from activation of IKKα and the non-canonical NF-κB pathway (63) and for others the relationship to non-canonical NF-κB was not explored. Thus, NIK modulates melanoma survival and growth through a β-catenin-mediated transcription way (64), is recruited to the promoters of pro-inflammatory genes to induce H3K9 histone acetylation in response to TNFα (65) and may favor or repress Smac mimetic induced death depending on the cell context. NIK upregulation in response to Smac mimetics/TNF repressed apoptosis induced by this combination, likely by maintaining FLICE inhibitory protein (c-FLIP) levels to suppress caspase-8 activation. Thus, resistant cells were sensitized to cell death by NIK depletion. NIK was required for activation of both canonical and non-canonical NF-κB pathways but their relative contribution to the protective effect was not explored (66).

## Non-Canonical NF-κB Activation and Kidney Disease

There is little information on the occurrence and role of non-canonical NF-κB activation in kidney disease. Few studies have addressed the overall regulation of the pathway. However, a few reports have explored individual molecules participating in non-canonical NF-κB activation, frequently without exploring function.

In diabetic mice kidney cortex NIK and RelB are upregulated several fold and phosphorylation of IKK alpha was increased (67). Non-canonical NF-κB components were predominantly located in tubular epithelial cells (67). NIK overexpression in cultured human proximal tubular cells increased RelB/p52 nuclear levels and DNA-binding activity and expression of inflammatory cytokines such as IL-6, IL-8, and MCP1 (68). TRAF3 silencing also increased nuclear RelB/p52 and transcription of proinflammatory cytokines. AGEs increased NIK and nuclear RelB/p52 in cultured proximal tubular cells (68).

In human kidney graft biopsies with delayed graft function NIK was increased in proximal tubular, interstitial, and mesangial cells and was observed in nuclei. In pig ischemia-reperfusion tubular and glomerular NIK phosphorylation was increased as observed by immunohistochemistry. In cultured proximal tubular cells thrombin induced NIK phosphorylation (69). However, no functional study addressed the consequences of NIK phosphorylation.

RelB targeting by siRNA may protect mice against lethal kidney ischemia (70). Mice injected with RelB siRNA had lower serum creatinine, histological tissue injury, and TNF expression as compared to controls. Furthermore, RelB targeting increased survival (70).

In cultured proximal tubular cells, lentiviral small hairpin RNA (shRNA)-mediated knockdown of RelB, abrogated the excess apoptosis induced by TNF in combination with cisplatin. Thus, cells with targeted RelB exposed to TNF/cisplatin have the same apoptosis rate as cells treated only with cisplatin. RelB targeting protection from apoptosis was associated with phenotypic markers of epithelial-to-mesenchymal transition. A transcriptomics analysis disclosed that knockdown of RelB was associated with upregulation of Snai2 and Rho GTPases. Targeting Rho kinase prevented the protective action of RelB knockdown (71).

The uremic toxins *p*-cresylsulfate and indoxylsulfate increased NF-κB2 expression by 50–80% in cultured proximal tubular cells (72). However, whether this was associated with increased protein levels or the functional consequences of this observation for the tubular cell cytotoxicity or inflammatory response elicited by these toxins (73) were not explored.

## TWEAK and Non-Canonical NF-κB Activation in Kidney Disease

A sustained NF-κB activation, persistent for up to 24 h, was observed in tubular cells exposed to TWEAK, consistent with activation of the non-canonical pathway in addition to the already characterized canonical NF-κB activation (20) (Table 1). In this regard, in cultured renal tubular cells TWEAK increases nuclear RelB/p52 accumulation, RelB and p52 DNA-binding activity, and NIK- and RelB-dependent CCL21 and CCL19 expression (23). Nuclear RelB/p52 migration and CCL21/CCL19 expression peaked at 24 h and, thus, were delayed as compared to RelA nuclear migration and expression of canonical RelA-dependent genes such as MCP1 and RANTES that peak at 3 and 6 h, respectively. By contrast, parthenolide, which inhibits the degradation of IκBα and RelA nuclear translocation, did not prevent CCL21 upregulation (20, 23, 74). Furthermore, TWEAK administration *in vivo* to healthy mice resulted in nuclear translocation of RelB and p52 in tubular cells and in increased renal CCL21 expression. Conversely, neutralizing anti-TWEAK antibodies prevented both RelB/p52 accumulation and increased expression of CCL21 in mice with folic acid-induced AKI (20). CCL21 expression had been previously shown to be dependent on non-canonical NF-κB activation in non-renal cells (53). CCL21 is T-cell and fibrocyte chemotactic factor that plays a role in renal tubulointerstitial fibrosis (75, 76).

**Table 1:** **TWEAK actions on kidney cells involving NF-***κ***B activation and evidence for the role of canonical or non-canonical pathways**.

Cell type	Effect	Functional modulation	NF-κB pathway involved	Reference
Mesangial cells	Inflammation	BAY11-7082	Canonical	Gao et al. (18)
Podocytes	Inflammation	Parthenolide	Canonical	Sanchez-Nino et al. (19)
Tubular cells	Inflammation	Parthenolide	Canonical	Sanz et al. (20)
	Proliferation	Parthenolide		Sanz et al. (9)
	Inflammation: CCL21, CCL19	NIK siRNA, RelB siRNA	Non-canonical	Sanz et al. (23)
Renal fibroblasts	Inflammation	Parthenolide	Canonical	Ucero et al. (22)

In summary, TWEAK is the only cytokine known to activate the non-canonical NF-κB pathway in tubular cells, both in cell culture and *in vivo*. Activation of the non-canonical NF-κB pathway is a key difference with TNF. However, whether TWEAK activates the non-canonical NF-κB pathway in mesangial cells, podocytes, or kidney fibroblasts and the functional in these cells remains unexplored.

## Conclusion

Accumulating evidence suggests a role for TWEAK in the pathogenesis of diverse forms of kidney injury, thus making TWEAK an attractive therapeutic target. Indeed, ongoing clinical trials are targeting TWEAK in kidney disease. Recently, a phase I clinical trial of anti-TWEAK neutralizing antibodies in rheumatoid arthritis was completed.^1^ Intravenous administration of anti-TWEAK resulted in undetectable serum-TWEAK for a month and in decreased levels of several inflammatory biomarkers. An ongoing phase II trial in lupus nephritis patients is testing the nephroprotective effect of BIIB023 anti-TWEAK antibody.^2^ TWEAK is one of a handful of cytokines that activate the non-canonical NF-κB pathway and the only one to have been explored with respect to non-canonical NF-κB pathway activation in kidney cells. Functional studies suggest that non-canonical NF-κB activation is a relevant action for TWEAK-induced kidney inflammation. Potential therapeutic approaches include both the simultaneous inhibition of both NF-κB pathways when targeting TWEAK as well as the eventual independent regulation of canonical and non-canonical NF-κB responses by designing differential inhibitors. While these non-canonical NF-κB inhibitors are not yet ready for human use, progress is being made on the design of NIK inhibitors (77). However, there is little functional information on the overall role of NIK and non-canonical NF-κB activation in kidney disease and on the consequences of differential therapeutically manipulation of canonical and non-canonical NF-κB responses. Clearly, more research is needed in this area.

## Author Contributions

Maria D. Sanchez-Niño and Alberto Ortiz devised the structure and overviewed and directed the effort. Luis C. Tabara, Jonay Poveda, Beatriz Fernandez-Fernandez, and Catalina Martin-Cleary reviewed the TWEAK and the non-canonical NF-κB literature, respectively. Ana B. Sanz and Rafael Selgas contributed to the final form.

## Conflict of Interest Statement

The authors declare that the research was conducted in the absence of any commercial or financial relationships that could be construed as a potential conflict of interest.

## Footnotes

^1^http://clinicaltrials.gov/ct2/show/NCT00771329

^2^http://clinicaltrials.gov/ct2/show/NCT01499355

## References

[1] Kidney Disease: Improving Global Outcomes (KDIGO) Acute Kidney Injury Work Group KDIGO clinical practice guideline for acute kidney injury. Kidney Int Suppl (2012) 2:1–138 10.1038/kisup.2012.3

[2] Kidney Disease: Improving Global Outcomes (KDIGO) CKD Work Group KDIGO 2012 clinical practice guideline for the evaluation and management of chronic kidney disease. Kidney Int Suppl (2013) 3:1–150 10.1038/kisup.2012.73

[3] Fernandez FB Elewa U Sanchez-Nino MD Rojas-Rivera JE Martin-Cleary C Egido J 2012 update on diabetic kidney disease: the expanding spectrum, novel pathogenic insights and recent clinical trials. Minerva Med (2012) 103:219–34 22805616

[4] Sanz AB Sanchez-Nino MD Ortiz A TWEAK, a multifunctional cytokine in kidney injury. Kidney Int (2011) 80:708–18 10.1038/ki.2011.180 21697814

[5] Sanz AB Sanchez-Nino MD Martin-Cleary C Ortiz A Ramos AM Progress in the development of animal models of acute kidney injury and its impact on drug discovery. Expert Opin Drug Discov (2013) 8:879–95 10.1517/17460441.2013.793667 23627598

[6] Chicheportiche Y Bourdon PR Xu H Hsu YM Scott H Hession C TWEAK, a new secreted ligand in the tumor necrosis factor family that weakly induces apoptosis. J Biol Chem (1997) 272:32401–10 10.1074/jbc.272.51.32401 9405449

[7] He F Dang W Saito K Watanabe S Kobayashi N Guntert P Solution structure of the cysteine-rich domain in Fn14, a member of the tumor necrosis factor receptor superfamily. Protein Sci (2009) 18:650–6 10.1002/pro.49 19241374 PMC2760370

[8] Brown SA Richards CM Hanscom HN Feng SL Winkles JA The Fn14 cytoplasmic tail binds tumour-necrosis-factor-receptor-associated factors 1, 2, 3 and 5 and mediates nuclear factor-kappaB activation. Biochem J (2003) 371:395–403 10.1042/BJ20021730 12529173 PMC1223299

[9] Sanz AB Sanchez-Nino MD Izquierdo MC Jakubowski A Justo P Blanco-Colio LM Tweak induces proliferation in renal tubular epithelium: a role in uninephrectomy induced renal hyperplasia. J Cell Mol Med (2009) 13:3329–42 10.1111/j.1582-4934.2009.00766.x 19426154 PMC4516489

[10] Justo P Sanz AB Sanchez-Nino MD Winkles JA Lorz C Egido J Cytokine cooperation in renal tubular cell injury: the role of TWEAK. Kidney Int (2006) 70:1750–8 10.1038/sj.ki.5001866 17003819

[11] Ortiz A Lorz C Catalan MP Danoff TM Yamasaki Y Egido J Expression of apoptosis regulatory proteins in tubular epithelium stressed in culture or following acute renal failure. Kidney Int (2000) 57:969–81 10.1046/j.1523-1755.2000.00925.x 10720950

[12] Nakayama M Ishidoh K Kojima Y Harada N Kominami E Okumura K Fibroblast growth factor-inducible 14 mediates multiple pathways of TWEAK-induced cell death. J Immunol (2003) 170:341–8 12496418 10.4049/jimmunol.170.1.341

[13] Vince JE Chau D Callus B Wong WW Hawkins CJ Schneider P TWEAK-FN14 signaling induces lysosomal degradation of a cIAP1-TRAF2 complex to sensitize tumor cells to TNFalpha. J Cell Biol (2008) 182:171–84 10.1083/jcb.200801010 18606850 PMC2447903

[14] Martin CC Moreno JA Fernandez B Ortiz A Parra EG Gracia C Glomerular haematuria, renal interstitial haemorrhage and acute kidney injury. Nephrol Dial Transplant (2010) 25:4103–6 10.1093/ndt/gfq493 20709744

[15] Kaplan MJ Lewis EE Shelden EA Somers E Pavlic R McCune WJ The apoptotic ligands TRAIL, TWEAK, and Fas ligand mediate monocyte death induced by autologous lupus T cells. J Immunol (2002) 169:6020–9 12421989 10.4049/jimmunol.169.10.6020

[16] Nakayama M Kayagaki N Yamaguchi N Okumura K Yagita H Involvement of TWEAK in interferon gamma-stimulated monocyte cytotoxicity. J Exp Med (2000) 192:1373–80 10.1084/jem.192.9.1373 11067885 PMC2193363

[17] Campbell S Burkly LC Gao HX Berman JW Su L Browning B Proinflammatory effects of TWEAK/Fn14 interactions in glomerular mesangial cells. J Immunol (2006) 176:1889–98 16424220 10.4049/jimmunol.176.3.1889

[18] Gao HX Campbell SR Burkly LC Jakubowski A Jarchum I Banas B TNF-like weak inducer of apoptosis (TWEAK) induces inflammatory and proliferative effects in human kidney cells. Cytokine (2009) 46:24–35 10.1016/j.cyto.2008.12.001 19233685

[19] Sanchez-Nino MD Poveda J Sanz AB Mezzano S Carrasco S Fernandez-Fernandez B Fn14 in podocytes and proteinuric kidney disease. Biochim Biophys Acta (2013) 1832:2232–43 10.1016/j.bbadis.2013.08.010 23999007

[20] Sanz AB Justo P Sanchez-Nino MD Blanco-Colio LM Winkles JA Kreztler M The cytokine TWEAK modulates renal tubulointerstitial inflammation. J Am Soc Nephrol (2008) 19:695–703 10.1681/ASN.2007050577 18235096 PMC2390965

[21] Linkermann A Brasen JH Darding M Jin MK Sanz AB Heller JO Two independent pathways of regulated necrosis mediate ischemia-reperfusion injury. Proc Natl Acad Sci U S A (2013) 110:12024–9 10.1073/pnas.1305538110 23818611 PMC3718149

[22] Ucero AC Benito-Martin A Fuentes-Calvo I Santamaria B Blanco J Lopez-Novoa JM TNF-related weak inducer of apoptosis (TWEAK) promotes kidney fibrosis and Ras-dependent proliferation of cultured renal fibroblast. Biochim Biophys Acta (2013) 1832:1744–55 10.1016/j.bbadis.2013.05.032 23748045

[23] Sanz AB Sanchez-Nino MD Izquierdo MC Jakubowski A Justo P Blanco-Colio LM TWEAK activates the non-canonical NFkappaB pathway in murine renal tubular cells: modulation of CCL21. PLoS One (2010) 5:e8955 10.1371/journal.pone.0008955 20126461 PMC2813291

[24] Hotta K Sho M Yamato I Shimada K Harada H Akahori T Direct targeting of fibroblast growth factor-inducible 14 protein protects against renal ischemia reperfusion injury. Kidney Int (2011) 79:179–88 10.1038/ki.2010.379 20927042

[25] Xia Y Campbell SR Broder A Herlitz L Abadi M Wu P Inhibition of the TWEAK/Fn14 pathway attenuates renal disease in nephrotoxic serum nephritis. Clin Immunol (2012) 145:108–21 10.1016/j.clim.2012.08.008 22982296 PMC3483377

[26] Izquierdo MC Sanz AB Mezzano S Blanco J Carrasco S Sanchez-Nino MD TWEAK (tumor necrosis factor-like weak inducer of apoptosis) activates CXCL16 expression during renal tubulointerstitial inflammation. Kidney Int (2012) 81:1098–107 10.1038/ki.2011.475 22278019

[27] Lu J Szeto CC Tam LS Lai FM Li EK Chow KM Relationship of intrarenal gene expression and the histological class of lupus nephritis – a study on repeat renal biopsy. J Rheumatol (2012) 39:1942–7 10.3899/jrheum.120177 22896023

[28] Lu J Kwan BC Lai FM Choi PC Tam LS Li EK Gene expression of TWEAK/Fn14 and IP-10/CXCR3 in glomerulus and tubulointerstitium of patients with lupus nephritis. Nephrology (Carlton) (2011) 16:426–32 10.1111/j.1440-1797.2011.01449.x 21303425

[29] Schwartz N Su L Burkly LC Mackay M Aranow C Kollaros M Urinary TWEAK and the activity of lupus nephritis. J Autoimmun (2006) 27:242–50 10.1016/j.jaut.2006.12.003 17257812

[30] Schwartz N Rubinstein T Burkly LC Collins CE Blanco I Su L Urinary TWEAK as a biomarker of lupus nephritis: a multicenter cohort study. Arthritis Res Ther (2009) 11:R143 10.1186/ar2816 19785730 PMC2787265

[31] Xuejing Z Jiazhen T Jun L Xiangqing X Shuguang Y Fuyou L Urinary TWEAK level as a marker of lupus nephritis activity in 46 cases. J Biomed Biotechnol (2012) 2012:359647 10.1155/2012/359647 22719208 PMC3375113

[32] El-Shehaby A Darweesh H El-Khatib M Momtaz M Marzouk S El-Shaarawy N Correlations of urinary biomarkers, TNF-like weak inducer of apoptosis (TWEAK), osteoprotegerin (OPG), monocyte chemoattractant protein-1 (MCP-1), and IL-8 with lupus nephritis. J Clin Immunol (2011) 31:848–56 10.1007/s10875-011-9555-1 21691937

[33] Moreno JA Izquierdo MC Sanchez-Nino MD Suarez-Alvarez B Lopez-Larrea C Jakubowski A The inflammatory cytokines TWEAK and TNFalpha reduce renal klotho expression through NFkappaB. J Am Soc Nephrol (2011) 22:1315–25 10.1681/ASN.2010101073 21719790 PMC3137579

[34] Zhao Z Burkly LC Campbell S Schwartz N Molano A Choudhury A TWEAK/Fn14 interactions are instrumental in the pathogenesis of nephritis in the chronic graft-versus-host model of systemic lupus erythematosus. J Immunol (2007) 179:7949–58 18025243 10.4049/jimmunol.179.11.7949

[35] Munoz-Garcia B Moreno JA Lopez-Franco O Sanz AB Martin-Ventura JL Blanco J Tumor necrosis factor-like weak inducer of apoptosis (TWEAK) enhances vascular and renal damage induced by hyperlipidemic diet in ApoE-knockout mice. Arterioscler Thromb Vasc Biol (2009) 29:2061–8 10.1161/ATVBAHA.109.194852 19778942

[36] Jakubowski A Ambrose C Parr M Lincecum JM Wang MZ Zheng TS TWEAK induces liver progenitor cell proliferation. J Clin Invest (2005) 115:2330–40 10.1172/JCI23486 16110324 PMC1187931

[37] Girgenrath M Weng S Kostek CA Browning B Wang M Brown SA TWEAK, via its receptor Fn14, is a novel regulator of mesenchymal progenitor cells and skeletal muscle regeneration. EMBO J (2006) 25:5826–39 10.1038/sj.emboj.7601441 17124496 PMC1698888

[38] Sanz AB Sanchez-Nino MD Ramos AM Moreno JA Santamaria B Ruiz-Ortega M NF-kappaB in renal inflammation. J Am Soc Nephrol (2010) 21:1254–62 10.1681/ASN.2010020218 20651166

[39] Moynagh PN The NF-kappaB pathway. J Cell Sci (2005) 118:4589–92 10.1242/jcs.02579 16219681

[40] Hoffmann A Natoli G Ghosh G Transcriptional regulation via the NF-kappaB signaling module. Oncogene (2006) 25:6706–16 10.1038/sj.onc.1209933 17072323

[41] Hayden MS Ghosh S Shared principles in NF-kappaB signaling. Cell (2008) 132:344–62 10.1016/j.cell.2008.01.020 18267068

[42] Perkins ND Integrating cell-signalling pathways with NF-kappaB and IKK function. Nat Rev Mol Cell Biol (2007) 8:49–62 10.1038/nrm2083 17183360

[43] Hayden MS Ghosh S Signaling to NF-kappaB. Genes Dev (2004) 18:2195–224 10.1101/gad.1228704 15371334

[44] Karin M Ben-Neriah Y Phosphorylation meets ubiquitination: the control of NF-[kappa]B activity. Annu Rev Immunol (2000) 18:621–63 10.1146/annurev.immunol.18.1.621 10837071

[45] Senftleben U Cao Y Xiao G Greten FR Krahn G Bonizzi G Activation by IKKalpha of a second, evolutionary conserved, NF-kappa B signaling pathway. Science (2001) 293:1495–9 10.1126/science.1062677 11520989

[46] Karin M How NF-kappaB is activated: the role of the IkappaB kinase (IKK) complex. Oncogene (1999) 18:6867–74 10.1038/sj.onc.1203219 10602462

[47] Tergaonkar V NFkappaB pathway: a good signaling paradigm and therapeutic target. Int J Biochem Cell Biol (2006) 38:1647–53 10.1016/j.biocel.2006.03.023 16766221

[48] Gilmore TD Introduction to NF-kappaB: players, pathways, perspectives. Oncogene (2006) 25:6680–4 10.1038/sj.onc.1209954 17072321

[49] Scheidereit C IkappaB kinase complexes: gateways to NF-kappaB activation and transcription. Oncogene (2006) 25:6685–705 10.1038/sj.onc.1209934 17072322

[50] Xiao G Harhaj EW Sun SC NF-kappaB-inducing kinase regulates the processing of NF-kappaB2 p100. Mol Cell (2001) 7:401–9 10.1016/S1097-2765(01)00187-3 11239468

[51] Xiao G Fong A Sun SC Induction of p100 processing by NF-kappaB-inducing kinase involves docking IkappaB kinase alpha (IKKalpha) to p100 and IKKalpha-mediated phosphorylation. J Biol Chem (2004) 279:30099–105 10.1074/jbc.M401428200 15140882

[52] Dejardin E The alternative NF-kappaB pathway from biochemistry to biology: pitfalls and promises for future drug development. Biochem Pharmacol (2006) 72:1161–79 10.1016/j.bcp.2006.08.007 16970925

[53] Dejardin E Droin NM Delhase M Haas E Cao Y Makris C The lymphotoxin-beta receptor induces different patterns of gene expression via two NF-kappaB pathways. Immunity (2002) 17:525–35 10.1016/S1074-7613(02)00423-5 12387745

[54] Coope HJ Atkinson PG Huhse B Belich M Janzen J Holman MJ CD40 regulates the processing of NF-kappaB2 p100 to p52. EMBO J (2002) 21:5375–85 10.1093/emboj/cdf542 12374738 PMC129074

[55] Claudio E Brown K Park S Wang H Siebenlist U BAFF-induced NEMO-independent processing of NF-kappa B2 in maturing B cells. Nat Immunol (2002) 3:958–65 10.1038/ni842 12352969

[56] Kayagaki N Yan M Seshasayee D Wang H Lee W French DM BAFF/BLyS receptor 3 binds the B cell survival factor BAFF ligand through a discrete surface loop and promotes processing of NF-kappaB2. Immunity (2002) 17:515–24 10.1016/S1074-7613(02)00425-9 12387744

[57] Novack DV Yin L Hagen-Stapleton A Schreiber RD Goeddel DV Ross FP The IkappaB function of NF-kappaB2 p100 controls stimulated osteoclastogenesis. J Exp Med (2003) 198:771–81 10.1084/jem.20030116 12939342 PMC2194184

[58] Saitoh T Nakayama M Nakano H Yagita H Yamamoto N Yamaoka S TWEAK induces NF-kappaB2 p100 processing and long lasting NF-kappaB activation. J Biol Chem (2003) 278:36005–12 10.1074/jbc.M304266200 12840022

[59] Rowe AM Murray SE Raue HP Koguchi Y Slifka MK Parker DC A cell-intrinsic requirement for NF-kappaB-inducing kinase in CD4 and CD8 T cell memory. J Immunol (2013) 191:3663–72 10.4049/jimmunol.1301328 24006459 PMC3815446

[60] Hofmann J Mair F Greter M Schmidt-Supprian M Becher B NIK signaling in dendritic cells but not in T cells is required for the development of effector T cells and cell-mediated immune responses. J Exp Med (2011) 208:1917–29 10.1084/jem.20110128 21807870 PMC3171087

[61] Sheng L Zhou Y Chen Z Ren D Cho KW Jiang L NF-kappaB-inducing kinase (NIK) promotes hyperglycemia and glucose intolerance in obesity by augmenting glucagon action. Nat Med (2012) 18:943–9 10.1038/nm.2756 22581287 PMC3766969

[62] Choudhary S Sinha S Zhao Y Banerjee S Sathyanarayana P Shahani S NF-kappaB-inducing kinase (NIK) mediates skeletal muscle insulin resistance: blockade by adiponectin. Endocrinology (2011) 152:3622–7 10.1210/en.2011-1343 21846802 PMC3176647

[63] Hacker H Chi L Rehg JE Redecke V NIK prevents the development of hypereosinophilic syndrome-like disease in mice independent of IKKalpha activation. J Immunol (2012) 188:4602–10 10.4049/jimmunol.1200021 22474019 PMC3532048

[64] Thu YM Su Y Yang J Splittgerber R Na S Boyd A NF-kappaB inducing kinase (NIK) modulates melanoma tumorigenesis by regulating expression of pro-survival factors through the beta-catenin pathway. Oncogene (2012) 31:2580–92 10.1038/onc.2011.427 21963849 PMC3253179

[65] Chung S Sundar IK Hwang JW Yull FE Blackwell TS Kinnula VL NF-kappaB inducing kinase, NIK mediates cigarette smoke/TNFalpha-induced histone acetylation and inflammation through differential activation of IKKs. PLoS One (2011) 6:e23488 10.1371/journal.pone.0023488 21887257 PMC3160853

[66] Cheung HH St Jean M Beug ST Lejmi-Mrad R LaCasse E Baird SD SMG1 and NIK regulate apoptosis induced by Smac mimetic compounds. Cell Death Dis (2011) 2:e146 10.1038/cddis.2011.25 21490678 PMC3122057

[67] Starkey JM Haidacher SJ LeJeune WS Zhang X Tieu BC Choudhary S Diabetes-induced activation of canonical and noncanonical nuclear factor-kappaB pathways in renal cortex. Diabetes (2006) 55:1252–9 10.2337/db05-1554 16644679

[68] Zhao Y Banerjee S LeJeune WS Choudhary S Tilton RG NF-kappaB-inducing kinase increases renal tubule epithelial inflammation associated with diabetes. Exp Diabetes Res (2011) 2011:192564 10.1155/2011/192564 21869881 PMC3159020

[69] Loverre A Ditonno P Crovace A Gesualdo L Ranieri E Pontrelli P Ischemia-reperfusion induces glomerular and tubular activation of proinflammatory and antiapoptotic pathways: differential modulation by rapamycin. J Am Soc Nephrol (2004) 15:2675–86 10.1097/01.ASN.0000139932.00971.E4 15466272

[70] Feng B Chen G Zheng X Sun H Zhang X Zhang ZX Small interfering RNA targeting RelB protects against renal ischemia-reperfusion injury. Transplantation (2009) 87:1283–9 10.1097/TP.0b013e3181a1905e 19424026

[71] Benedetti G Fokkelman M Yan K Fredriksson L Herpers B Meerman J The nuclear factor kappaB family member RelB facilitates apoptosis of renal epithelial cells caused by cisplatin/tumor necrosis factor alpha synergy by suppressing an epithelial to mesenchymal transition-like phenotypic switch. Mol Pharmacol (2013) 84:128–38 10.1124/mol.112.084053 23625948

[72] Sun CY Hsu HH Wu MS p-Cresol sulfate and indoxyl sulfate induce similar cellular inflammatory gene expressions in cultured proximal renal tubular cells. Nephrol Dial Transplant (2013) 28:70–8 10.1093/ndt/gfs133 22610984

[73] Poveda J Sanchez-Niño MD Glorieux G Sanz AB Egido J Vanholder R p-Cresyl sulphate has pro-inflammatory and cytotoxic actions on human proximal tubular epithelial cells. Nephrol Dial Transplant (2013). [Epub ahead of print]. 10.1093/ndt/gft367 24166466

[74] Hehner SP Hofmann TG Droge W Schmitz ML The antiinflammatory sesquiterpene lactone parthenolide inhibits NF-kappa B by targeting the I kappa B kinase complex. J Immunol (1999) 163:5617–23 10553091

[75] Sakai N Wada T Yokoyama H Lipp M Ueha S Matsushima K Secondary lymphoid tissue chemokine (SLC/CCL21)/CCR7 signaling regulates fibrocytes in renal fibrosis. Proc Natl Acad Sci U S A (2006) 103:14098–103 10.1073/pnas.0511200103 16966615 PMC1599918

[76] Carragher D Johal R Button A White A Eliopoulos A Jenkinson E A stroma-derived defect in NF-kappaB2-/- mice causes impaired lymph node development and lymphocyte recruitment. J Immunol (2004) 173:2271–9 15294939 10.4049/jimmunol.173.4.2271

[77] Li K McGee LR Fisher B Sudom A Liu J Rubenstein SM Inhibiting NF-kappaB-inducing kinase (NIK): discovery, structure-based design, synthesis, structure-activity relationship, and co-crystal structures. Bioorg Med Chem Lett (2013) 23:1238–44 10.1016/j.bmcl.2013.01.012 23374866

